# A year at the forefront of plasmodesmal biology

**DOI:** 10.1242/bio.060123

**Published:** 2023-10-24

**Authors:** Andrea Paterlini

**Affiliations:** Institute of Molecular Plant Sciences, School of Biological Sciences, University of Edinburgh, Edinburgh EH9 3BF, UK

**Keywords:** Development, Evolution, Hormones, Plants, Plasmodesmata, Signalling, Stress

## Abstract

Cell–cell communication is a central feature of multicellular organisms, enabling division of labour and coordinated responses. Plasmodesmata are membrane-lined pores that provide regulated cytoplasmic continuity between plant cells, facilitating signalling and transport across neighboring cells. Plant development and survival profoundly depend on the existence and functioning of these structures, bringing them to the spotlight for both fundamental and applied research. Despite the rich conceptual and translational rewards in sight, however, the study of plasmodesmata poses significant challenges. This Review will mostly focus on research published between May 2022 and May 2023 and intends to provide a short overview of recent discoveries, innovations, community resources and hypotheses.

## Teaser

**Summary:** This Review provides a short overview of recent discoveries, innovations, community resources and hypotheses in the field of plasmodesmata biology. It mostly focuses on research published between May 2022 and May 2023.

*What's past is prologue*, William Shakespeare, The Tempest

## Introduction

**Fig. 1.**
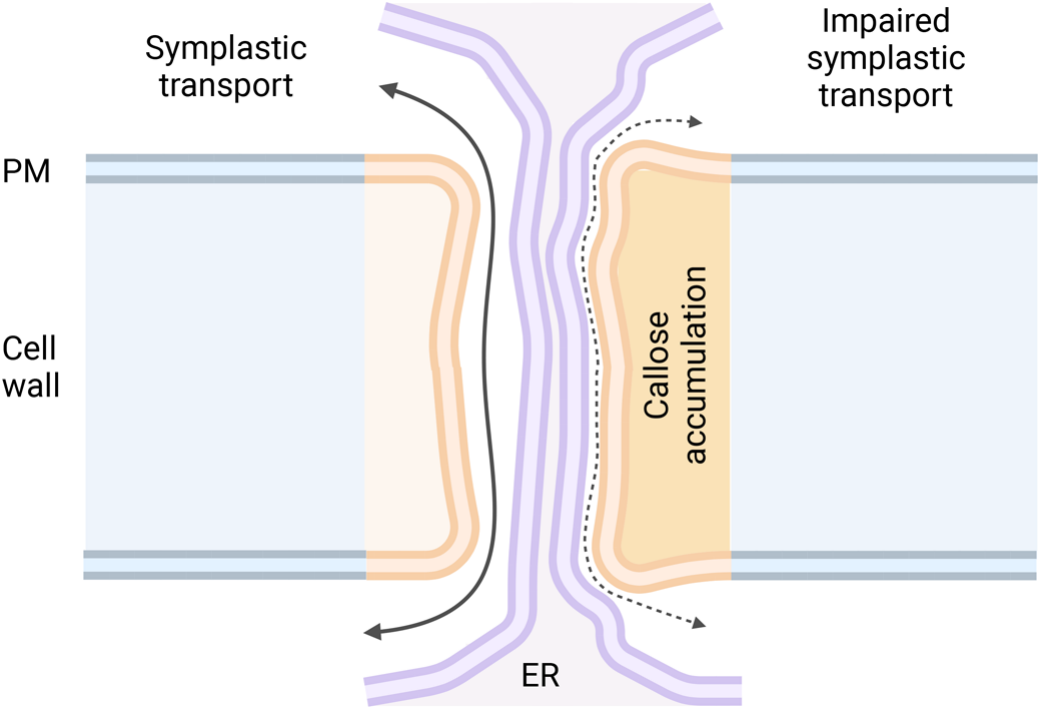
**Structure and regulation of plasmodesmata (PD).** PD are membrane-lined pores spanning the wall between neighbouring plant cells. A constricted form of the endoplasmic reticulum (ER) – termed the desmotubule – is present within each pore (rendered in purple in the diagram). The space between the two PD membranes, called the cytoplasmic sleeve, represents the main route for symplastic transport across PD. The lipids and polysaccharides present in the plasma membrane (PM) and proximal wall of PD (shades of orange) differ from the rest of the cellular membrane and wall (shades of blue). The diagram displays two spatially and temporally separate PD conformations: on the left, PD are ‘open’ and conducive to cell–cell transport; on the right, PD are ‘closed’ and transport is heavily restricted. Callose deposition in the PD proximal wall (darker orange) is likely to mediate this reversible change by pushing the PM against the ER and restricting the available space for transport (Radford et al., 1998). Membrane deformation might occur in the process. Additional mechanisms (not shown for simplicity) can contribute to PD permeability changes (Li et al., 2021). The image was created using BioRender.com.

“*Plasmodesmata are small pores connecting neighbouring plant cells*.” This sentence will sound familiar (and perhaps a bit trite) to the many researchers in our field. However, growing numbers of new researchers are becoming fascinated by plasmodesmata (PD). Some actively seek this challenging field for its many open questions (Schreier et al., 2022 preprint); others delve into it by coincidence, discovering that these structures are involved in processes of interest to them (Nicolas et al., 2022). Encounters of this second type are hardly surprising when one appreciates the central transport and signalling roles of these structures. These plasma membrane (PM)-lined pores put in direct contact the cytosol of most plant cells, creating symplastic routes for diffusion and bulk flow of nutrients and signals. Continuity of the endomembrane system [via a constricted form of the endoplasmic reticulum (ER)] is also uniquely present (Fig. 1). PD can also act as sites for local signalling and responses (Li et al., 2021).

The mixing of different backgrounds and interests brought by scientists engaging with PD has ultimately yielded, I believe, a diverse and welcoming community. No matter the route through which scientists approach it, their contributions to PD biology are valued. Established and emerging leaders in PD biology have been recently featured in several journal interviews (Burch-Smith, 2022; Benitez-Alfonso, 2022; Barberon, 2022; Faulkner, 2020).

In this brief piece, I will review recent developments in the field. I hope it will be a helpful summary for novices and experts alike. As it is simply not possible to cover all relevant papers, please view this as a very personal selection. Absence of mention does not at all indicate lack of significance.

### Discoveries

Humans – scientists included – are visual creatures drawn to weird and wonderful things. The aerial potatoes reported by Nicolas et al. (2022) represent the most striking PD-associated phenotype of the year. *BRANCHED1* encodes a transcription factor negatively regulating shoot branching. In potato, a paralog of this gene seems to additionally prevent tuber formation in above-ground parts of the plant. This function within shoot axillary buds is, in part, mediated by regulating components of abscisic acid (ABA) signalling. This plant hormone has been shown to influence callose, a polysaccharide that occludes PD, preventing transport across the same (Fig. 1) (Tylewicz et al., 2018). Delivery of resources and tuber-inducing factors to buds is ultimately prevented (Nicolas et al., 2022). Note that the permeability of PD can also be affected by mechanisms other than callose (Li et al., 2021).

Interplays between hormones and PD are further highlighted by Mehra et al. (2022). Plants repress lateral root formation in the presence of dry soil pockets, and a switch in hydraulic fluxes can explain this behaviour in *Arabidopsis thaliana*. While water normally flows from the root surface into internal tissues, in areas of low humidity, the flow is reversed. Outward water movement from the vasculature carries ABA with it. Movement of the hormone via PD is assumed, although not fully demonstrated. Were it to be the case, it would be another example of the significance of passive hormone transport (Paterlini, 2020). ABA can then locally transcriptionally upregulate factors stimulating PD callose deposition (Fig. 1). Symplastic movement of auxin, a critical hormone for lateral root development, is in turn compromised, suppressing branching (Mehra et al., 2022).

The relevance of auxin transport via PD has been previously reviewed (Paterlini, 2020; Band, 2021). Linh and Scarpella (2022) expand this by addressing *A. thaliana* leaf vein patterning. A passive auxin transport component resolves previous modelling and experimental gaps (Ravichandran et al., 2020). At early stages, auxin is shown to canalize its own movement within (proto)vein cells, by regulating the permeability of local PD. While reciprocal feedbacks with active transport and signalling are evident, the molecular mechanisms remain to be clarified (Linh and Scarpella, 2022).

Precursors to brassinosteroid (BR) hormones have also just been shown to traffic across PD in *A. thaliana* (Wang et al., 2023). Experimental closure of PD significantly altered the status of BR-responsive transcription factors. Movement of precursors across PD was also directly monitored with biorthogonal chemistry. BRs, in turn, could regulate PD, promoting callose deposition in treated roots (Fig. 1). This feedback is likely to be transcriptional in nature (Xiong et al., 2022; Nolan et al., 2023) and might carefully balance hormone transport. The significance of this movement, however, is currently unclear: phenotypes were mild (Wang et al., 2023) and potentially not solely influenced by impaired BR movement.

### Technological innovations

**Fig. 2.**
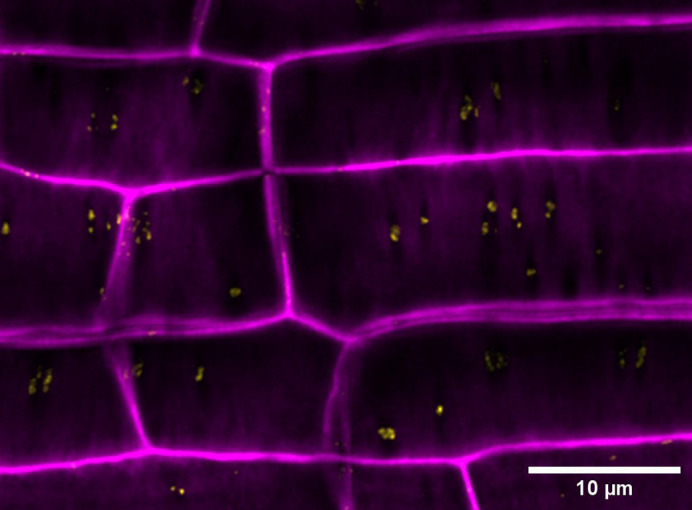
**PD subcellular domains have unique molecular compositions.** Clusters of PD are visible at the interface between epidermal and cortex cell layers in the root of an *Arabidopsis thaliana* seedling*.* PD are identified by the presence of the Multiple C2 domains and transmembrane region protein 4 (MCTP4), false coloured in yellow. MCTP4 is enriched at PD and is involved in ER–PM contact sites (Brault et al., 2019). Propidium iodine – false coloured in magenta – is used to stain polysaccharide components of the cell wall. Stain signal around PD is significantly lower than that in other regions: the PD proximal wall has a different polysaccharide composition. The author collected a *z*-stack of confocal images from the *MCTP:MCTP4-GFP* line. Stacks were processed with deconvolution software and a standard deviation projection was generated. Brightness and contrast of the final image were adjusted to make the pattern clearly visible on screens.

PD are spatially defined by the repertoire of lipids, polysaccharides and proteins present in the local membranes and proximal walls (Figs 1 and 2). These components influence both the structure and function of PD (Li et al., 2021). Studying these components (and PD in general), however, poses significant methodological challenges. The 2nd edition of the ‘*Plasmodesmata: Methods and Protocols*’ book (Benitez-Alfonso and Heinlein, 2022) represents an active effort to support new practitioners in the field. The past year also provided us with valuable innovations and applications.

The presence of individual polysaccharides in the proximal wall of PD can be assessed with specific antibodies or stains (Fig. 2). Paterlini et al. (2022), instead, exploited subcellular fractionation and enzymatic fingerprinting to characterize entire polysaccharide classes associated with PD in *A. thaliana* cell cultures. A high abundance of rhamnogalacturonan I (RG-I) pectins was observed with this approach. In a separate study, Okawa et al. (2023) showed that loss of arabinogalactan proteins (AGPs) unexpectedly altered the permeability of *A. thaliana* PD. The close association between AGP and RG-I (Tan et al., 2023), and the abundance of the latter at PD (Paterlini et al., 2022), suggest that these glycoproteins might also be local, important PD components.

PD-located proteins (PDLPs) promote PD closure during biotic and abiotic interactions (Mehra et al., 2022; Tee et al., 2023). Data from Li et al. (2022 preprint) seem to indicate that constitutive overexpression of (selected) PDLP members only results in callose accumulation within the different, native domains of expression of the same. As PDLPs lack intrinsic enzymatic activity (Thomas et al., 2008), the presence/absence of relevant protein partners could gate callose accumulation. The composition of PD might therefore be cell-type specific. Li et al. (2022 preprint) employed biotin proximity labelling to identify specific PDLP-interacting proteins in *A. thaliana*. This is the first example of proximity labelling targeted to PD, and the approach holds promise to resolve local signalling complexes. However, it is important to still remember that proteins enriched at PD are not necessarily solely localized there (Brault et al., 2019).

Mechanisms targeting proteins to PD have not been fully clarified. Luna et al. (2023) highlight the role of unconventional amino acid stretches located on the extracellular portion of PD proteins. Identification of these segments, due to low sequence conservation, was specifically aided by machine-learning algorithms developed for the purpose. Recognition processes for these signals remain to be addressed.

### New resources

Had evolution taken a different route other than the repeated emergence of PD in the green lineage (Brunkard and Zambryski, 2017; Paterlini, 2020), our field of research would simply not exist. It might therefore surprise readers from different disciplines that the formation of PD, arguably the most central question, remains an unresolved process. New community resources will be necessary to tackle this question.

Species performing C4 photosynthesis have a higher density of PD at specific interfaces of their leaves (to facilitate the necessary metabolic fluxes and carbon-concentrating mechanisms) (Danila et al., 2016). Schreier et al. (2022 preprint) provide data supporting the idea that photosynthesis itself is the cue triggering such densities in *Gynandropsis gynandra*. While the paper does not resolve PD formation, its central resource role lies in the establishment of a system (organism and stimulus) to monitor the process under a range of chemical treatments and, in a foreseeable future, genetic backgrounds (a genome for *G. gynandra* is already available; Hoang et al., 2023).

Hess et al. (2022) provided a phylogenomic analysis of *Zygnematophyceae*, the sister clade to land plants. The common ancestor of the two clades might have been multicellular, implying a (potential) loss of that state during the evolution of *Zygnematophyceae*. Present species are unicellular or filamentous. Mining their genomes (and comparing them to those of land plants) might identify important genes for PD formation (as suggested by Keller and Delaux, 2022). Additionally, species with the ‘intermediate’ filamentous growth (Hess et al., 2022) could be an ideal platform for PD (re-)introduction (provided that species become genetically tractable).

Formation and function of PD depend on proteins enriched in this cellular domain (Fig. 2) (Li et al., 2021). PD proteomes are therefore valuable tools for reverse genetic studies. Johnston et al. (2023) and Gombos et al. (2023), together, provide one PD proteome from mature *A. thaliana* leaves and two from the moss *Physcomitrium patens*. Moss PD proteomes are significant, as they are the first ones from a non-flowering plant. Taking advantage of orthologous genes, the authors probe relationships within gene families: the evolution of PD association seems to follow variable patterns.

Differences in PD proteomes between (and within) species clearly exist (Johnston et al., 2023; Li et al., 2022 preprint; Gombos et al., 2023). An endless quest for different PD lists might not be advisable. Kirk et al. (2022) sidestep this issue by developing a computational tool predicting PD proteomes in species that have not been experimentally sampled. Expression datasets and interaction networks of interest can then be superimposed on candidate lists in targeted studies. The legume *Medicago truncatula* and the process of nodulation are used as an example.

### New hypotheses

Identifying a suitable biological question is a key challenge for scientists trying to establish their own research programme. The field of PD, due to its numerous unsolved aspects, is an amenable ground for inquisitive scientists. Some potentials new streams of enquiry are described below.

As discussed, PD are unique cellular subdomains, both in terms of architecture and composition. New fluorescent reporters recently revealed pH differences in *A. thaliana* cell subdomains (Moreau et al., 2022). As conditions in the PD apoplast might affect the activity of wall-remodelling enzymes (Paterlini et al., 2022), an assessment with these reporters is highly enticing. Super-resolution microscopy (Fitzgibbon et al., 2010) will likely be required in the process.

Novel functions might also be assigned to PD. A mechano-sensitive ion channel was shown to function at ER–PM membrane contact sites in *A. thaliana* cells (Codjoe et al., 2022). PD, in reason of the close apposition between different membranes, are considered a specialized contact site (Figs 1 and 2) (Tilsner et al., 2016). A rapid screen of a PD proteome (Brault et al., 2019) reveals that at least one related mechanosensitive ion channel could be present. Being at the junction between two cells, PD would be ideal sites for the detection of forces and stresses. As PD mechano-sensing was already explored (via modelling) in Park et al. (2019), the field seems ripe for a detailed investigation.

Functional parallels can also be drawn with other organisms. Septal pores between some fungal cells – despite independent evolution – structurally resemble PD and can be similarly occluded in response to stimuli (Bloemendal and Kuck, 2013). Mamun et al. (2023) undertook a large-scale identification of proteins involved in the plugging process. Interestingly, one such protein localized at the pore and possessed a lipid-binding C2 domain. This structural feature is shared with PD proteins critical for ER–PM membrane tethering and PD function (Fig. 2) (Brault et al., 2019; Ishikawa et al., 2020). Insights on convergent evolution between plants and fungi might soon emerge.

Lastly, previous evidence had suggested that PD were involved in the propagation of calcium waves upon tissue wounding (Toyota et al., 2018). Those experiments had employed plants with constitutively open/closed PD, and drastic changes on conductivity can produce pleiotropic effects. Using more refined inducible lines, Bellandi et al. (2022) show that PD connectivity is not required for calcium spread. Propagation of wound signals seems to occur in the apoplast, rather than the symplast (Bellandi et al., 2022; Gao et al., 2023). Clarifying processes in which PD are not involved can be as relevant as exploring those in which they are.

## Future prospects

PD research directly feeds into many important questions facing plant science (Armstrong et al., 2023). A thorough understanding of plant signalling and transport holds transformative potential for our food systems. Modified PD could ensure growth robustness in the face of biotic and abiotic challenges. Altered resource/signal allocation to specific parts of a plant could shape yields and the consumption of agricultural inputs.

Knowledge gaps around PD formation and function hold back these appealing applications. Research will also need to be carried out (or translated) into crop species. Many ambitious goals are beyond our individual careers. Nurturing the next generations of PD scientists is therefore the most important legacy for the community. We should also get solace from the incremental nature of science: past and present discoveries – big or small as they may feel – might be the basis of a PD-driven revolution of tomorrow. Together, we are writing the prologue to an exciting future story.
